# Exploring Autonomic Alterations during Seizures in Temporal Lobe Epilepsy: Insights from a Heart-Rate Variability Analysis

**DOI:** 10.3390/jcm12134284

**Published:** 2023-06-26

**Authors:** Sung-Min You, Baek-Hwan Cho, Hyo-Eun Bae, Young-Kyun Kim, Jae-Rim Kim, Soo-Ryun Park, Young-Min Shon, Dae-Won Seo, In-Young Kim

**Affiliations:** 1 Department of Biomedical Engineering, Hanyang University, Seoul 04763, Republic of Korea; seungmin@hanyang.ac.kr; 2 Fetal Neonatal Neuroimaging and Developmental Science Center, Boston Children’s Hospital, Harvard Medical School, Boston, MA 02115, USA; 3 Department of Biomedical Informatics, School of Medicine, CHA University, Seongnam 13488, Republic of Korea; baekhwan.cho@cha.ac.kr; 4 Institute of Biomedical Informatics, School of Medicine, CHA University, Seongnam 13488, Republic of Korea; 5 Department of Neurology, Samsung Medical Center, Sungkyunkwan University School of Medicine, Seoul 06351, Republic of Koreasonogung@gmail.com (Y.-M.S.); 6 Neuroscience Center, Samsung Medical Center, Seoul 06351, Republic of Korea

**Keywords:** epilepsy, temporal lobe epilepsy, heart-rate variability, autonomic regulation, seizure

## Abstract

Epilepsy’s impact on cardiovascular function and autonomic regulation, including heart-rate variability, is complex and may contribute to sudden unexpected death in epilepsy (SUDEP). Lateralization of autonomic control in the brain remains the subject of debate; nevertheless, ultra-short-term heart-rate variability (HRV) analysis is a useful tool for understanding the pathophysiology of autonomic dysfunction in epilepsy patients. A retrospective study reviewed medical records of patients with temporal lobe epilepsy who underwent presurgical evaluations. Data from 75 patients were analyzed and HRV indices were extracted from electrocardiogram recordings of preictal, ictal, and postictal intervals. Various HRV indices were calculated, including time domain, frequency domain, and nonlinear indices, to assess autonomic function during different seizure intervals. The study found significant differences in HRV indices based on hemispheric laterality, language dominancy, hippocampal atrophy, amygdala enlargement, sustained theta activity, and seizure frequency. HRV indices such as the root mean square of successive differences between heartbeats, pNN50, normalized low-frequency, normalized high-frequency, and the low-frequency/high-frequency ratio exhibited significant differences during the ictal period. Language dominancy, hippocampal atrophy, amygdala enlargement, and sustained theta activity were also found to affect HRV. Seizure frequency was correlated with HRV indices, suggesting a potential relationship with the risk of SUDEP.

## 1. Introduction

Epilepsy affects 6.38 per 1000 persons worldwide and is known as one of the most functionally distressful neurologic diseases [1,2]. Many studies have investigated the underlying complexity of the connection between the brain and the heart in patients with epilepsy [3]. Cardiovascular autonomic regulation is impaired in patients with epilepsy, as manifested by impaired heart-rate variability (HRV) in response to various stimuli or reduced uptake of 123I-metaiodobenzylguanidine, a marker of postganglionic sympathetic dysfunction [4,5,6,7].

Although the mechanism responsible for autonomic dysfunction in epilepsy patients has yet to be fully elucidated, alterations in cardiac ion channel expression and the detrimental effects of cortical epileptic discharge on centrally mediated cardiac outputs constitute the suggested pathophysiology [8]. Abnormal epileptic discharges in the brain can also affect central brain regions that regulate autonomic activity and produce these cardiac symptoms, either at the onset or during the propagation of a seizure [9].

These autonomic alterations are related to cardiorespiratory disturbances, such as tachycardia, asystole, and even sudden unexpected death in epilepsy (SUDEP) [9,10,11,12,13]. Excessive activation of the sympathetic nervous system due to intense seizures may contribute to cardiopulmonary dysfunction [14] and prolonged suppression of brain activity [15], which can cause impaired arousal when the heart functions independently. Intense seizures may also cause compensative responses, such as an elevated adenosine level, and these responses can contribute to sudden death [16].

Previous studies have reported that a higher seizure frequency is associated with an increased risk of SUDEP [17,18,19], but the mechanisms underlying this relationship remain unclear and require further investigation. Several proposed hypotheses include cumulative metabolic and cardiovascular stress associated with frequent seizures, neuronal damage, dysfunction, and altered autonomic control [20,21]. More precisely, abrupt sympathetic activation triggered by seizures can lead to cardiopulmonary and brainstem dysfunction related to apnea and arousal, potentially contributing to SUDEP [14].

Although the specific pathophysiological mechanism is not clear, some studies imply that most cases of SUDEP are due to postictal central respiratory dysfunction, which eventually causes cardiac arrest [16,22,23]. Because SUDEP represents a major cause of premature deaths in patients with epilepsy, an understanding of the effect of seizures on cardiovascular functions is needed [24].

The role of cerebral hemispheric lateralization in the regulation of cardiac autonomic control continues to be a topic of considerable debate and ongoing research [25]. The key controversy surrounds the representation of parasympathetic and sympathetic functions within the dominant and nondominant hemispheres. There is growing evidence to suggest that the dominant hemisphere, typically the left hemisphere for right-handed individuals, is predominantly associated with parasympathetic function, while the nondominant hemisphere is linked with sympathetic representation. Previous studies with brain stimulation have tried to show that the dominant hemisphere is associated with parasympathetic function, while the nondominant hemisphere is associated with sympathetic representation. For example, some studies have found that stimulating the left insula produces parasympathetic effects while stimulating the right insula produces sympathetic effects [26]. Others have found increased sympathetic activity and decreased vagal tone after inactivating the left hemisphere with intracarotid amobarbital [27,28]. Likewise, studies of ischemic lesions indicate that acute left hemisphere stroke has been found to reduce cardiac vagal tone, whereas right hemispheric stroke or epilepsy may lead to prominent parasympathetic deficits against left lateralization of vagal tone, [29] and similar lateralization was observed in seven patients with focal insular stroke [30]. 

The lateralization of cortical autonomic efferent and afferent representations is still the subject of debate since these findings have not been universally observed, and some studies have reported differing outcomes [31,32]. Some studies suggest that parasympathetic representations are located in the dominant hemisphere, while sympathetic representations are located in the nondominant hemisphere [25]. Likewise, there are inconsistencies in the literature devoted to human lesions, and previous studies have been limited by small sample sizes and variations in clinical context and methodology. Therefore, further investigation is needed to clarify the role of cerebral hemispheric lateralization in cardiac autonomic control.

The Wada test is considered one of the best ways to determine dominant hemisphere laterality, although previous studies using intracarotid amobarbital tests have produced inconsistent results. In mesial temporal epilepsy involving the hippocampus and amygdala, autonomic ictal manifestations are common, and pathologic laterality in temporal lobe epilepsy (TLE) can be confirmed by magnetic resonance imaging (MRI) of abnormalities such as hippocampal sclerosis and amygdala enlargement. The ictal discharges of sustained rhythmic theta activities have been identified as important biomarkers of mesial TLE with high lateralized values.

Analysis of HRV is one of the most accurate indicators of sympathovagal balance in the autonomic nervous system (ANS) [33]. Recently, there has been increased interest in monitoring HRV in epileptic patients [34] due to the association between dysregulation of the cardiac autonomic nervous system and long-term morbidity and mortality in epileptic patients [34,35]. HRV monitoring can be utilized to elucidate this pathophysiology and to develop preventive measures for SUDEP [36,37].

Conventional HRV analysis requires a long-term heart-rate sequence, such as 24 h of measurements. Long-term HRV is useful in assessments of the homeostatic mechanisms related to circadian rhythms, core body temperature, sleep cycles, and general body metabolisms of the body and has been described as the gold standard of clinical HRV assessment [38]. Long-term HRV analysis based on 24 h measurements has been used to predict the risks of acute myocardial infarction and chronic heart failure [39,40]. On the other hand, short-term HRV analysis, which can be performed with shorter sequences of heart rates (5 min), has proved to be useful and reliable in various fields since 1996 [41,42,43]. A short-term HRV analysis can help focus on the effects of interactions between sympathetic and parasympathetic branches of the ANS and respiratory sinus arrhythmia rather than the effect of general circadian periodicity [44]. Short-term HRV analysis has also been used in epileptic patients. Several studies used short-term HRV analysis to investigate the cardiac alterations around ictal onsets in epileptic patients [45,46,47,48].

Recently, several types of research showed the possibility of HRV analysis with a shorter interval of recordings. This approach is called ultra-short-term (UST) HRV analysis, which refers to analysis with intervals of less than 5 min. Several researchers investigated the reliability of UST HRV analysis. Salahuddin et al. [49] used 24 healthy subjects to show that 60 s is enough time to estimate HRV parameters, and Nussinovitch et al. [50,51] investigated the minimum length of heat-rate sequences required to estimate HRV parameters that are computed with 5 min intervals. They found that a 60 s interval was sufficient to approximate most HRV parameters. Other studies [52,53,54] also found that UST HRV analysis can serve as a useful surrogate method to assess HRV. The objective of this study was to assess the differences in autonomic patterns within each interval (preictal, ictal, postictal) along with the laterality and clinical factors in TLE patients using UST HRV analysis.

## 2. Materials and Methods

### 2.1. Materials

In this study, researchers retrospectively reviewed the medical records of the Samsung Medical Center Epilepsy Monitoring Unit between 2014 and 2020. The patients had been diagnosed with temporal lobe epilepsy by reviewing presurgical noninvasive evaluation data, and an epilepsy conference was conducted to plan an invasive study. All of the patients had undergone presurgical noninvasive evaluations, including a scalp video-electroencephalogram of habitual seizures, brain MRI scans with 3.0 Tesla MRI, positron emission tomography scans using 18F-fluorodeoxyglucose, ictal single-photon emission computed tomography scans with subtracted 99m-Tc ethyl cysteine dimer, and a complete neuropsychological examination. After these studies were reviewed in multidisciplinary intensive epilepsy conferences, the type of epilepsy and further therapeutic strategies were identified by reviewing presurgical noninvasive evaluation data.

Patients were selected from the database if they met the following criteria: (1) definite TLE with electrophysiologic, semiologic, and imaging findings, (2) the presence of ictal tachycardia > 100 bpm or > 20% increase in baseline heart rate, (3) identifiable QRS complexes on ictal electrocardiogram (ECG), (4) a history of a focal seizure without secondary generalization, and (5) a history of intervals longer than 180 s between ictal events. Patients who had probable psychological paroxysmal events were excluded from this study. The authors retrospectively selected patients with TLE from the medical records following these criteria. Data were anonymized by removing direct identifiers from the dataset and were also deidentified before analysis. The authors included 75 patients (36 males and 39 females) between the ages of 12 and 71 years (mean age: 36.3 ± 13.5 years). A total of 41 patients had left TLE and 34 had right TLE. 

The authors selected ECG recordings of seizure events that are suitable for HRV analysis from these patients. During their hospitalization in the epilepsy monitoring unit, the use of antiepileptic drugs was suspended to inspect ictal symptoms during seizures. The electroclinical data were reviewed separately by two epileptologists (Seo, D.W., Shon, Y.M.), who determined and marked the onset and end of seizures based on intracranial EEG to define the ictal interval. The authors used invasive EEG to define the precise interval of seizures for the ictal recording analysis. The start (onset) and end (offset) of ictal periods were defined by analyzing both invasive ictal recordings that show clear EEG waveform without artifacts and the corresponding video. Dr. Seo and Dr. Shon repeatedly reviewed both videos and invasive recordings by marking ictal intervals until they reach their agreement. The authors analyzed the ECG signals from three intervals. The first interval was the preictal period, which is defined as the three minutes before seizure onset. The second interval, the postictal period, is defined as the three minutes after the seizure offset. Last, the ictal interval is the period of each seizure. Demographic and clinical data of the subjects are described in Table 1. This study was approved by the Institutional Review Board of Samsung Medical Center (IRB No. 2019-12-104-003, 8 January 2021) and was conducted according to the principles of the Declaration of Helsinki. As this was a retrospective study using electronic medical records, the requirement for individual informed consent from patients was waived by the Institutional Review Board.

### 2.2. HRV Data Acquisition and Preprocessing

The authors collected ECG data using a NicoletOne LTM system (Natus Medical Incorporated, Pleasanton, CA, USA) at a sampling rate of 512 Hz. The signals were recorded with two electrodes on the shoulders to form the lead 1 channel of ECG. The authors filtered the ECG signals to remove various noises related to patients’ movements, breathing, and muscle electrical activity. All subsequent processing was performed within MATLAB 2020 (The MathWorks, Inc., Natick, MA, USA). First, the authors applied a third-order Butterworth filter with a high-pass 0.5 Hz cutoff to eliminate baseline wander. Then, the authors used a notch filter with a 60 Hz stop band to clear out powerline interference [55]. Lastly, the authors performed low-pass filtering with a moving-average filter with a 20 Hz cutoff to filter other artifacts such as electromyograms from the ECG signals [56]. From the filtered ECG signal, the authors extracted the R peak with a wavelet transform–based QRS complex detector [57] and computed the corresponding RR intervals for each heartbeat. Since a single ectopic beat could distort the result in UST HRV analysis, ectopic beats were detected and excluded. The authors removed ectopic beats using a method based on the HRV signal [58]; a heartbeat was considered ectopic if there was a change of beat-to-beat interval (RRi) 20% over the average 5 beats before and after. Clinicians reviewed every record of ECG after filtering and ectopic peak removal to confirm the reliability of the detected R points, removed ectopic beats, and final RRi sequences.

### 2.3. Ultra-Short-Term HRV Analysis

Although the three intervals were defined as preictal, ictal, and postictal, the ictal duration could be different for each seizure. For HRV analysis, the authors used the central 60 s of ECG recordings from each interval to match the length of the segment [48].

The time domain, frequency domain, and nonlinear HRV indices were extracted for each interval [33]. For the time-domain HRV indices, the authors used the mean value of the RR interval (mean RRi), the standard deviation of all RR intervals (SDNN), the root mean square of the successive differences (RMSSD), and the percentage of absolute differences in successive NN values > 50 ms (pNN50) [59].

For the frequency domain HRV indices, the authors applied Lomb–Scargle (LS) periodograms to calculate the power spectral density of the HRV [60]. The LS periodogram is a method of spectral analysis for unevenly sampled data. Previous research has demonstrated that LS periodograms are more appropriate than other spectral methods that require resampling for short-term spectral analysis. LS periodograms can mitigate errors due to missing data, including portions missing due to corruption or ectopy [61,62]. The spectral powers of the heart rate were divided into two components: low frequency (LF, 0.04–0.15 Hz) and high frequency (HF, 0.15–0.4 Hz). The power of each band was normalized by the ratio of the total power of the heart-rate spectrum, except for the very low-frequency band (<0.04 Hz). In general, the LF band is known to be influenced by both sympathetic and vagal activity, whereas the HF band represents only vagal activity [63,64,65]. To figure out the effect of the parasympathetic activity in LF spectral power and to provide an indicator of sympathovagal balance, the LF/HF ratio was calculated [66,67].

The researchers also computed nonlinear HRV indices, the cardiovagal index (CVI) and cardiosympathetic index (CSI), which are derived from a Poincare plot of the RRi series [68,69]. These indices provide complementary information about the sympathetic and parasympathetic influence on HRV.

### 2.4. Statistical Analysis

The authors performed statistical analyzes using Stata, version 15 (StataCorp. 2017. Stata Statistical Software: Release 15. College Station, TX, USA: StataCorp LLC). Data normality was checked using a kurtosis and skewness test [70]. A *p*-value < 0.05 was considered statistically significant. First, the authors used the independent t-test to compare the HRV parameters in each interval to assess the differences between the left and right TLE groups. Then, the authors used repeated measures analyses of variance (ANOVAs) to group differences in longitudinal HRV changes for the three intervals (preictal, ictal, postictal) as the within-subjects factor and laterality as the between-subjects factor. As a post hoc test, the authors applied the Tukey–Kramer multiple comparisons tests (alpha = 0.05) [71]. The authors also analyzed HRV indices for four subgroups: patients with or without language dominancy, hippocampal atrophy, amygdala enlargement, or sustained theta activity. In subgroup analysis, the nonparametric Mann–Whitney U-test was performed as those subsets did not satisfy normality [72].

## 3. Results

HRV indices vary in individuals with different neurological conditions. This study aimed to investigate the differences in HRV indices based on hemispheric lateralization, language dominancy, hippocampal atrophy, amygdala enlargement, sustained theta activity, and seizure frequency.

### 3.1. Lateral Difference of HRV Alteration around the Ictal Period

Independent t-tests revealed that most HRV indices showed statistical differences in laterality in the ictal interval. Among the time-domain HRV indices, RMSSD and pNN50 showed significant differences (*p* < 0.001). For the frequency-domain HRV indices, normalized LF, normalized HF, and LF/HF ratio were significantly different in the ictal interval (*p* < 0.001). Furthermore, the CVI also showed significant differences (*p* = 0.006) (Table 2).

Repeated-measures ANOVAs showed that the mean RRi and CVI differed statistically only by laterality (*p* = 0.023 for mean RRi, *p* = 0.036 for RMSSD). However, those two parameters did not significantly interact with the interval. The interaction between laterality and interval was statistically significant for RMSSD (*p* = 0.006), pNN50 (*p* < 0.001), normalized LF (*p* = 0.017), normalized HF (*p* < 0.001), and LF/HF (*p* < 0.001) (Table 3).

### 3.2. Effects of Neurological Clinical Factors on HRV Alteration

The results of the study showed that language dominancy had a significant effect on HRV indices during the ictal period. The time-domain HRV indices RMSSD and pNN50 differed significantly (*p* = 0.015 and *p* < 0.001, respectively), along with the frequency-domain HRV indices of normalized LF, normalized HF, and LF/HF ratio (*p* = 0.046, *p* < 0.001, and *p* = 0.001, respectively) (Table 4).

Further analysis of the data with the Mann–Whitney U-test revealed that hippocampal atrophy was associated with differences in HRV indices during the preictal and ictal intervals. The normalized HF during the preictal interval and mean RRi and RMSSD during the ictal interval differed significantly between the two groups (z = −2.44, *p* = 0.015; z = −2.09, *p* = 0.037; and z = −2.45, *p* = 0.015, respectively).

In addition, an enlarged amygdala was also found to affect HRV indices during the ictal period. Mann–Whitney U-tests found that the RMSSD and normalized LF during the ictal period differed significantly between the two groups (z = 2.33, *p* = 0.020; and z = −2.16, *p* = 0.031, respectively).

Sustained theta activity was also found to have an impact on HRV indices. The CVI during the ictal interval showed significant differences between the two groups (z = 2.20, *p* = 0.028), while during the postictal interval, normalized HF and the LF/HF ratio differed significantly between the two groups (z = 3.05, *p* = 0.002; and z = −2.7, *p* = 0.007, respectively). This could be related to elevated parasympathetic (vagal) activity during the postictal period [54].

Finally, the study also identified a correlation between seizure frequency and HRV indices. The normalized LF and LF/HF ratio during the ictal interval were positively correlated with seizure frequency (r[49] = 0.31, *p* = 0.025; and r[49] = 0.32, *p* = 0.022, respectively), while the LF/HF ratio during the postictal period showed a negative correlation with seizure frequency (r[49] = −0.29, *p* = 0.041). This could be related to the underlying relationship between seizure frequency (or severity) and the risk of SUDEP.

## 4. Discussion

The study found that the HRV indices varied significantly during the ictal interval by laterality, with significant differences in RMSSD, pNN50, normalized LF, normalized HF, and LF/HF ratio.

### 4.1. Ictal Cardiac Manifestation along with Hemispheric Lateralization

**Figure 1:**
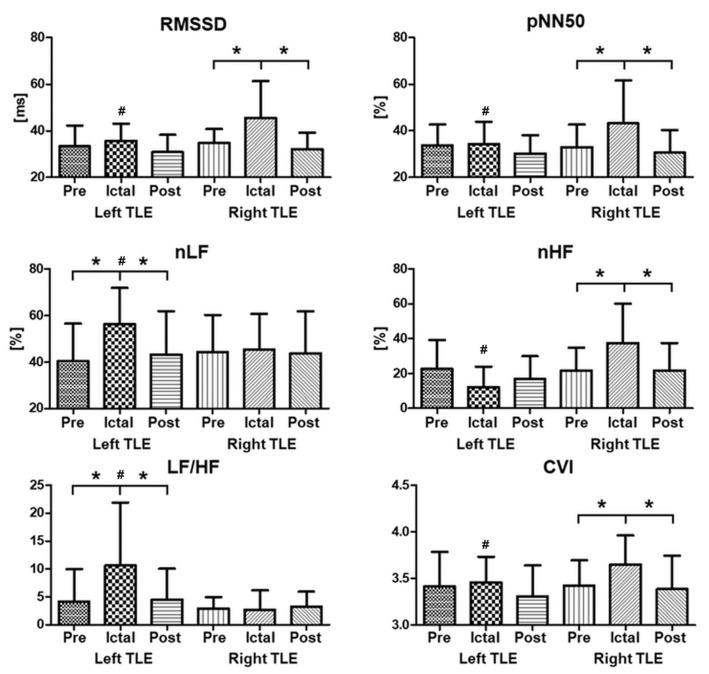
Comparison of HRV indices along with laterality and intervals. * Significantly different between intervals (preictal, postictal); # significantly different along with laterality (left–right); all significant results from Tukey–Kramer multiple comparison tests.

Most of the time- and frequency-domain HRV parameters showed significant alterations in the ictal interval (Table 2 and Figure 1). Among the time-domain HRV indices, decreases in mean RRi were observed during the ictal interval, indicating ictal tachycardia as a common symptom. Increases in RMSSD and pNN50 were also found during the ictal interval. In a prior study, RMSSD and pNN50 were higher during the interictal state in patients with epilepsy compared with normal controls [73]. The RMSSD indicates beat-to-beat variance in the heart rate, which is related to vagally mediated changes [38].

In an examination of the influence of laterality, various indices of HRV demonstrated different aspects during the ictal period, as depicted in Figure 1. Regarding laterality, the study found that most HRV indices included statistical differences between the right and left sides during the ictal period. These results are consistent with those of previous studies [48,54,74], which also reported lateral differences in HRV indices in patients with TLE. Both normalized low-frequency (nLF) power and the LF/HF ratio exhibited substantial increases during the ictal interval, indicating higher sympathetic activity. The amygdala, which is highly interconnected with hypothalamic and brainstem autonomic structures, plays a crucial role in complex autonomic disturbances in the temporal lobe. An augmented sympathovagal state during the ictal period can be attributed to the contiguity of the epileptogenic zones in TLE to adjacent limbic structures, including the amygdala, hippocampus, and insula, which form part of the central autonomic network [75]. Previous research has documented sympathetic dominance features in the interictal state [34,64,76]. However, patients with right-sided TLE exhibited significant increases in the RMSSD, pNN50, nHF, and CVI during the ictal interval.

Controversies about the lateral dominance of sympathetic and parasympathetic activities in the central autonomic system are well documented. This result is not consistent with the left lateralization of vagal tone found in controls, showing elevated HRV indices related to vagal tone in right-TLE patients. The central autonomic system has wide and complex connections within the limbic system, where ictal activities can cause autonomic dysfunction in the form of both activation and disturbance. The previous finding about the lateralized dominance of vagal tone in controls cannot be easily interpreted as it created an expectation for increased vagal tone in left-sided TLE patients. In epilepsy patients, the balanced autonomic network between hemispheres can be disrupted during epileptic seizures, causing an autonomic disturbance on the right hemispheric network in right-sided TLE patients, which eventually causes a relative increment of vagal activity in the left hemisphere.

### 4.2. Effects of Neurological Clinical Factors on HRV Alteration

The authors also found that language dominancy had a significant effect on HRV indices during ictal intervals, with significant differences in RMSSD, pNN50, normalized LF, normalized HF, and LF/HF ratio (Table 4, Table S1). The nondominant group showed increased RMSSD and pNN50 during ictal intervals, suggesting heightened parasympathetic activity during seizures. The parasympathetic nervous system is responsible for the “rest and digest” response and is associated with relaxation and recovery. These results suggest that individuals with nondominant hemispheric onset seizures may have a greater parasympathetic response during seizures. In contrast, the dominant group showed an increased LF/HF ratio during ictal intervals, which is an indicator of sympathetic activity. The sympathetic nervous system is known to be related to the “fight or flight” response and is associated with stress and increased arousal. The increased LF/HF ratio in the dominant language group suggests a heightened sympathetic response during seizures. These findings suggest that the origin of seizures, depending on hemispheric dominancy, may play a role in modulating ANS activity during seizures, with different patterns observed between dominant and nondominant groups.

In addition, hippocampal atrophy, amygdala enlargement, and sustained theta activity were found to have an impact on HRV indices during various intervals. Hippocampal atrophy refers to the shrinkage or loss of neurons in the hippocampus, a region of the brain involved in memory and emotion regulation. Previous studies reported a possible link between hippocampal atrophy and temporal epilepsy in clinical and pathological aspects [77,78] and those findings support this link in terms of autonomic alteration observed by UST HRV analysis (Table S2). Amygdala enlargement refers to an increase in the size or volume of the amygdala, a region of the brain involved in processing emotions. Previous studies reported the possibility of a connection between TLE and amygdala enlargement [79,80]; however, its effect on ANS is still unknown. In this study, a group with amygdala enlargement exhibited relatively strong parasympathetic activity during the ictal period, suggesting a connection between amygdala enlargement and ANS (Table S3). Sustained theta activity refers to the presence of abnormal theta rhythms in an EEG during interictal periods. Studies have shown that sustained theta activity is associated with reduced HRV during interictal periods, indicating a shift toward sympathetic dominance (Table S4).

The limitations of this study include the small sample size, lack of a control group, limited long-term monitoring, single-center study design, and potential confounding factors. These limitations may affect the generalizability, internal validity, and reliability of the findings. Therefore, broadened studies including a design with large populations, control-group comparisons, replication studies in different populations and settings based on multicenter design, longitudinal observations of HRV alterations over time, integrating multimodal approaches, investigation of the effects of interventions, and studies that enhance external validity are recommended for future works.

## 5. Conclusions

In this study, the authors investigated HRV alteration around seizures in TLE patients. The authors found that some HRV indices vary significantly during the ictal interval by laterality, suggesting hemispheric lateralization of ANS. This was demonstrated by significant differences in several indices, including RMSSD, pNN50, normalized LF, normalized HF, and LF/HF ratio. The authors also analyzed the HRV indices of patients based on various neurological clinical factors, such as language dominancy, hippocampal atrophy, amygdala enlargement, sustained theta activity, and seizure frequency. These findings suggest that neurological clinical factors can influence HRV indices during different phases of seizure in terms of HRV indices. Lastly, the authors also highlight the potential role of HRV indices in understanding the physiological changes occurring during seizures and their potential to be used as biomarkers for assessing epilepsy severity and the risk of SUDEP [81,82]. The authors showed that elevated sympathetic activity and/or demoted parasympathetic activity are related to seizure frequency, which could be a potential key to understanding the risk of SUDEP. This study provides valuable insights into HRV alterations during seizures in epilepsy patients, although several limitations need to be addressed. Future studies could provide a more comprehensive understanding of the role of the ANS in epilepsy and its associated alterations. Additionally, the ultra-short-term HRV analysis could be a way to investigate possible connections between cardiac alteration and clinical symptoms such as Mnonosomy 1p36 syndrome [83] or Moyamoya disease [84] in patients with epilepsy. This could have implications for potential therapeutic approaches and interventions to modulate autonomic function in epilepsy patients, ultimately improving their clinical outcomes.

## Figures and Tables

**Table 1:** Demographical and clinical information of patients in the study population.

	Total (*n* = 75)	Left TLE (*n* = 41)	Right TLE (*n* = 34)	*p*-Value *
Sex (M/F)	36/39	16/25	20/14	
Age at evaluation	36.3 ± 13.5	35.9 ± 13.8	36.9 ± 13.2	0.965
Age at seizure onset	21.4 ± 15.5	21.2 ± 15.4	14.7 ± 12.3	0.724
Disease duration (year)	14.9 ± 11.1	21.6 ± 15.9	15.2 ± 9.5	0.071
Etiology of epilepsy				0.730 **
Cryptogenic	17	11	6	
Hippocampal sclerosis	38	21	17	
Cortical malformation	4	1	3	
Tumor	6	2	4	
Vascular malformation	6	4	2	
History of encephalitis	2	1	1	
Destructive lesion	2	1	1	
Frequency of seizure				0.187 **
Daily	11	8	3	
Weekly	16	11	5	
Monthly	45	20	25	
Yearly	3	2	1	
Number of seizures	7.9 ± 6.2	9.1 ± 7.5	6.5 ± 3.6	0.020
Number of ASMs used	2.73 ± 1.4	2.7 ± 1.3	2.8 ± 1.5	0.443
Laterality (Rt/Lt/bilateral)				0.000 **
Rt	30	0	30	
Lt	33	33	0	
Bilateral or nonlateralized	12	8	4	

* *p*-value indicates the significant difference along with laterality; ** chi-square test; ASM: antiseizure medication.

**Table 2:** Independent *t*-tests of the HRV parameters in each interval.

HRV INDEX	PREICTAL			ICTAL			POSTICTAL		
	Left	Right	*p*	Left	Right	*p*	Left	Right	*p*
RRI (ms)	**751.0 ± 136.2**	**847.3 ± 202.2**	0.02 *	581.0 ± 90.5	595.4 ± 118.7	0.55	685.4 ± 131.0	703.0 ± 143.8	0.58
SDNN (ms)	108.6 ± 70.7	95.7 ± 37.7	0.34	104.7 ± 51.4	128.6 ± 72.0	0.10	87.2 ± 50.7	101.6 ± 65.9	0.29
RMSSD (ms)	33.3 ± 8.9	34.8 ± 6.0	0.41	**35.7 ± 7.4**	**45.6 ± 15.7**	<0.01 *	30.8 ± 7.4	32.2 ± 7.0	0.41
pNN50	6.5 ± 0.5	5.1 ± 1.7	0.14	**7.2 ± 3.7**	**13.5 ± 7.7**	<0.01 *	4.7 ± 3.7	5.5 ± 3.3	0.32
nLF (%)	40.3 ± 16.2	44.3 ± 15.9	0.28	**56.4 ± 15.5**	**45.4 ± 15.4**	<0.01 *	43.2 ± 18.7	43.7 ± 18.2	0.90
nHF (%)	22.6 ± 16.6	21.6 ± 13.2	0.78	**12.2 ± 11.8**	**37.6 ± 22.5**	<0.01 *	17.0 ± 13.0	21.8 ± 15.7	0.15
LF/HF	4.2 ± 5.8	3.0 ± 2.0	0.25	**10.6 ± 11.2**	**2.6 ± 3.5**	<0.01 *	4.5 ± 5.5	3.3 ± 2.6	0.24
CSI	3.4 ± 0.4	3.4 ± 0.3	0.94	**3.5 ± 0.3**	**3.6 ± 0.3**	0.01 *	3.3 ± 0.3	3.4 ± 0.4	0.33
CVI	6.0 ± 4.5	4.9 ± 2.2	0.19	5.7 ± 3.6	5.3 ± 2.9	0.60	5.0 ± 2.4	5.6 ± 3.0	0.35

* Values in bold indicate *p*-value < 0.05.

**Table 3:** Repeated measures ANOVA for the HRV parameters along with laterality and interval.

HRV Index	Source	$\eta_{p}^{2}$	F	*p*
Mean RRi (ms)	**Laterality**	**0.023**	**5.25**	0.023 *
	**Interval**	**0.280**	**42.59**	<0.001 *
	Laterality × interval	0.019	2.06	0.130
SDNN (ms)	Laterality	0.005	1.14	0.288
	**Interval**	**0.024**	**2.69**	0.070 *
	Laterality × interval	0.017	1.93	0.148
RMSSD (ms)	Laterality	**0.052**	**12.06**	<0.001 *
	**Interval**	**0.151**	**19.48**	<0.001 *
	**Laterality × interval**	**0.046**	**5.32**	0.006 *
pNN 50 (%)	Laterality	**0.435**	**9.97**	0.002 *
	**Interval**	**0.208**	**28.71**	<0.001 *
	**Laterality × interval**	**0.115**	**14.25**	<0.001 *
Normalized LF (%)	Laterality	0.004	0.93	0.336
	**Interval**	**0.050**	**5.76**	0.004 *
	**Laterality × interval**	**0.037**	**4.15**	0.017 *
Normalized HF (%)	**Laterality**	**0.090**	**21.54**	<0.001 *
	Interval	0.020	2.27	0.106
	**Laterality × interval**	**0.117**	**14.54**	<0.001 *
LF/HF ratio	**Laterality**	**0.074**	**17.60**	<0.001 *
	**Interval**	**0.048**	**5.52**	0.005 *
	**Laterality × interval**	**0.063**	**7.37**	<0.001 *
CVI	**Laterality**	**0.012**	**4.46**	0.036 *
	**Interval**	**0.068**	**7.96**	0.001 *
	Laterality × interval	0.014	1.52	0.221
CSI	Laterality	0.002	0.51	0.474
	Interval	0.001	0.11	0.896
	Laterality × interval	0.012	1.28	0.280

* $\eta_{p}^{2}$: effect size as partial eta square. Values in bold indicate *p*-value < 0.05.

**Table 4:** Repeated measures ANOVA for the HRV parameters between dominant and nondominant cases based on the Wada test.

HRV INDEX	PREICTAL			ICTAL			POSTICTAL		
–	Dominant (*n* = 26)	Nondominant (*n* = 32)	*p*	Dominant (*n* = 26)	Nondominant (*n* = 32)	*p*	Dominant (*n* = 26)	Nondominant (*n* = 32)	*p*
RRI (ms)	773.46 ± 99.4	829.9 ± 222.1	0.235	593.6 ± 87.7	570.2 ± 107.3	0.375	691.2 ± 95.2	683.9 ± 148.1	0.830
SDNN (ms)	120.0 ± 74.5	95.0 ± 41.4	0.112	107.6 ± 52.5	131.0 ± 75.3	0.184	97.8 ± 54.6	101.9 ± 68.5	0.803
RMSSD (ms)	34.5 ± 9.3	34.1 ± 6.7	0.876	**35.3 ± 7.8**	**44.1 ± 16.5**	0.015 *	31.7 ± 7.2	31.9 ± 7.5	0.914
pNN50	6.6 ± 4.6	5.0 ± 1.9	0.071	**7.7 ± 4.3**	**13.8 ± 7.9**	<0.001 *	5.3 ± 4.3	5.6 ± 3.5	0.802
nLF (%)	37.9 ± 14.6	42.4 ± 16.2	0.279	**57.0 ± 15.9**	**48.0 ± 17.3**	0.046 *	43.9 ± 17.5	44.5 ± 18.3	0.895
nHF (%)	24.9 ± 14.3	19.7 ± 14.0	0.169	**14.0 ± 14.0**	**36.5 ± 22.8**	<0.001 *	19.7 ± 14.4	22.2 ± 15.4	0.528
LF/HF	3.5 ± 6.3	3.9 ± 3.6	0.618	**9.7 ± 10.3**	**3.0 ± 4.0**	0.001 *	3.8 ± 3.2	3.2 ± 2.6	0.456
CSI	3.5 ± 0.3	3.4 ± 0.3	0.203	3.5 ± 0.3	3.6 ± 0.4	0.071	3.4 ± 0.3	3.4 ± 0.4	0.795
CVI	6.8 ± 5.1	5.0 ± 2.4	0.075	6.0 ± 3.8	5.6 ± 3.1	0.659	5.6 ± 2.5	5.6 ± 3.1	0.995

* Dominant: seizures with dominant hemispheric onset; nondominant: seizures with nondominant hemispheric onset. The bold values indicate a *p*-value less than 0.05.

## Data Availability

The data are not publicly available due to ethical restrictions.

## Supplementary Materials

The following supporting information can be downloaded at: https://www.mdpi.com/article/10.3390/jcm12134284/s1. Table S1. Repeated measures ANOVA for the HRV parameters along with language dominance and interval; Table S2. Independent t-test results for HRV parameters with hippocampal atrophy in each interval; Table S3. Independent t-test results for HRV parameters with amygdala enlargement in each interval; Table S4. Independent t-test for HRV parameters with sustained theta activity in each interval.

## Abbreviation

Mean RRi: mean of interbeat intervals between successive heartbeats; SDNN, standard deviation of the normal-to-normal interval; RMSSD, square root of the mean of the sum of the squares of differences between consecutive normal-to-normal intervals; nLF, relative power of the frequency band (0.04–0.15 Hz); nHF, relative power of the high-frequency band (0.15–0.4 Hz); LF/HF, ratio of low- to high-frequency power; CVI, cardio vagal index; CSI (cardiosympathetic index); EEG, electroencephalogram.
