# Supplementary material for: Exploring Autonomic Alterations during Seizures in Temporal Lobe Epilepsy: Insights from a Heart-Rate Variability Analysis
Source: J Clin Med. 2023 Jun 26;12(13):4284. doi: 10.3390/jcm12134284 (PMC10342618; doi:10.3390/jcm12134284)
Supplement: Supplementary file 1 [file jcm-12-04284-s001.zip › jcm-2407724-supplementary.pdf]

Supplementary Table S1. Repeated measures ANOVA for the HRV parameters along with language dominance and interval.

HRV index	Source	η_p^2	F	*p*
Mean RRi [ms]	Dominance	0.0010	0.17	0.6844
	**Interval**	**0.3003**	**36.04**	**0.0000**
	Dominance x interval	0.0156	1.33	0.2675
SDNN [ms]	Dominance	0.0001	0.01	0.9251
	Interval	0.0165	1.41	0.2467
	Dominance x interval	0.2528	2.18	0.1164
RMSSD [ms]	Dominance	0.0215	3.69	0.0564
	**Interval**	**0.1008**	**9.41**	**0.0001**
	**Dominance x interval**	**0.0439**	**3.85**	**0.0231**
pNN 50 [%]	Dominance	0.0266	4.59	0.0335
	**Interval**	**0.2057**	**21.76**	**0.0000**
	**Dominance x interval**	**0.1072**	**10.09**	**0.0001**
Normalized LF [%]	Dominance	0.0015	0.26	0.6138
	**Interval**	**0.0879**	**8.09**	**0.0004**
	Dominance x interval	0.0285	2.46	0.0884
Normalized HF [%]	**Dominance**	**0.0400**	**7.00**	**0.0089**
	Interval	0.0122	1.04	0.3568
	**Dominance x interval**	**0.1156**	**10.98**	**0.0000**
LF/HF ratio	**Dominance**	**0.0433**	**7.60**	**0.0065**
	**Interval**	**0.0580**	**5.17**	**0.0066**
	**Dominance x Interval**	**0.0782**	**7.12**	**0.0011**
CVI	Dominance	0.0002	0.03	0.8601
	Interval	0.0458	4.03	0.0194
	Dominance x interval	0.0273	2.36	0.0974
CSI	Dominance	0.0121	2.06	0.1530
	Interval	0.0013	0.11	0.8970
	Dominance x interval	0.0136	1.16	0.3160

Supplementary Table S2. Independent t-test results for HRV parameters with hippocampal atrophy in each interval.

HRV INDEX	PRE-ICTAL			ICTAL			POST-ICTAL		
	**Positive (n = 32)**	**Negative (n = 41)**	*p*	**Positive (n = 32)**	**Negative (n = 41)**	*p*	**Positive (n = 32)**	**Negative (n = 41)**	*p*
RRI [ms]	791.1 ± 204.6	793.9 ± 149.7	0.9480	558.8 ± 93.9	606.6 ± 108.6	0.0516	661.6 ± 127.0	713.6 ± 134.7	0.0973
SDNN [ms]	105.4 ± 69.9	98.8 ± 47.3	0.6352	117.2 ± 75.5	112.9 ± 50.9	0.7685	84.1 ± 46.1	100.5 ± 66.6	0.2397
RMSSD [ms]	34.2 ± 9.3	33.4 ± 6.0	0.6519	39.1 ± 17.3	41.3 ± 8.2	0.4696	**29.5 ± 5.6**	**32.9 ± 8.1**	**0.0454**
pNN50	5.7 ± 4.2	5.6 ± 3.3	0.8903	9.2 ± 6.0	10.8 ± 7.2	0.3083	4.7 ± 3.0	5.3 ± 4.0	0.5136
nLF [%]	42.1 ± 15.4	41.9 ± 17.1	0.9735	51.8 ± 17.4	50.8 ± 15.7	0.8011	44.6 ± 18.8	43.2 ± 17.9	0.7540
nHF [%]	**18.0 ± 13.3**	**25.7 ± 16.0**	**0.0309**	22.5 ± 21.6	25.5 ± 21.7	0.5622	17.9 ± 15.7	17.9 ± 13.7	0.4160
LF/HF	4.3 ± 4.1	3.1 ± 4.5	0.2543	7.2 ± 9.5	6.6 ± 9.7	0.7726	3.6 ± 3.0	4.4 ± 5.4	0.4241
CSI	3.4 ± 0.4	3.4 ± 0.3	0.9691	3.5 ± 0.4	3.6 ± 0.3	0.4423	3.3 ± 0.3	3.4 ± 0.4	0.1451
CVI	5.3 ± 2.9	5.7 ± 4.3	0.7128	5.8 ± 3.6	5.2 ± 2.9	0.4074	5.1 ± 2.8	5.3 ± 2.7	0.7233

Supplementary Table S3. Independent t-test results for HRV parameters with amygdala enlargement in each interval.

HRV INDEX	PRE-ICTAL			ICTAL			POST-ICTAL		
	**Positive (n = 14)**	**Negative (n = 61)**	***p***	**Positive (n = 14)**	**Negative (n = 61)**	***p***	**Positive (n = 14)**	**Negative (n = 61)**	***p***
RRI [ms]	725.0 ± 150.1	810.6 ± 177.4	0.0990	605.2 ± 120.1	583.5 ± 100.3	0.4834	680.5 ± 124.3	696.4 ± 139.7	0.6971
SDNN [ms]	103.8 ± 62.8	102.6 ± 57.5	0.9428	131.1 ± 94.7	111.94 ± 52.7	0.3024	98.3 ± 55.1	92.7 ± 59.2	0.7457
RMSSD [ms]	32.7 ± 7.9	34.3 ± 7.7	0.4837	**47.1 ± 21.6**	**38.6 ± 9.4**	**0.0238**	32.1 ± 6.8	31.3 ± 7.3	0.7021
pNN50	5.4 ± 3.9	6.0 ± 4.1	0.6476	12.6 ± 10.2	9.5 ± 5.5	0.1172	5.0 ± 2.2	5.1 ± 3.8	0.9218
nLF [%]	40.5 ± 22.6	42.5 ± 14.4	0.6718	**42.1 ± 16.2**	**53.5 ± 15.7**	**0.0175**	41.8 ± 17.6	43.8 ± 18.6	0.7214
nHF [%]	27.1 ± 20.6	21.0 ± 13.5	0.1810	29.0 ± 30.3	22.5 ± 19.1	0.3073	15.7 ± 12.9	20.0 ± 14.7	0.3205
LF/HF	4.4 ± 8.0	3.4 ± 3.3	0.4584	6.3 ± 7.8	7.2 ± 9.9	0.7708	4.2 ± 2.9	3.9 ± 4.8	0.8049
CSI	3.4 ± 0.3	3.4 ± 0.3	0.6598	3.6 ± 0.4	3.5 ± 0.3	0.2479	3.4 ± 0.4	3.3 ± 0.3	0.6962
CVI	6.5 ± 6.5	5.3 ± 2.7	0.2620	5.2 ± 3.5	5.6 ± 3.3	0.6663	5.6 ± 3.4	5.2 ± 2.5	0.5838

Supplementary Table S4. Independent t-test for HRV parameters with sustained theta activity in each interval.

HRV INDEX	PRE-ICTAL			ICTAL			POST-ICTAL		
	**Positive (n = 24)**	**Negative (n = 48)**	***p***	**Positive (n = 24)**	**Negative (n = 48)**	***p***	**Positive (n = 24)**	**Negative (n = 48)**	***p***
RRI [ms]	791.2 ± 186.2	798.4 ± 175.6	0.8722	587.3 ± 100.2	587.8 ± 109.6	0.9837	672.0 ± 130.5	711.2 ± 138.8	0.2533
SDNN [ms]	112.4 ± 74.0	97.1 ± 50.3	0.3061	135.8 ± 75.2	108.2 ± 54.0	0.0784	92.0 ± 70.8	95.5 ± 52.7	0.8147
RMSSD [ms]	35.2 ± 10.0	33.3 ± 6.5	0.3249	41.1 ± 18.9	40.3 ± 8.8	0.8154	31.2 ± 9.5	32.0 ± 6.1	0.7197
pNN50	5.7 ± 3.2	5.7 ± 4.3	0.9623	11.9 ± 8.3	9.4 ± 5.7	0.1346	5.1 ± 4.6	5.2 ± 3.0	0.9620
nLF [%]	42.4 ± 15.2	41.0 ± 16.5	0.7365	51.1 ± 17.9	51.9 ± 15.5	0.8547	44.9 ± 14.8	43.9 ± 19.7	0.8335
nHF [%]	25.3 ± 13.9	20.5 ± 16.0	0.2135	30.0 ± 23.8	21.3 ± 20.3	0.1113	**26.9 ± 14.2**	**16.3 ± 13.2**	**0.0026**
LF/HF	2.3 ± 1.8	4.3 ± 5.3	0.0750	4.3 ± 2.7	8.5 ± 11.1	0.0850	2.7 ± 3.0	4.5 ± 5.0	0.1136
CSI	3.5 ± 0.4	3.4 ± 0.3	0.3318	2.6 ± 0.3	3.5 ± 0.3	0.1581	3.3 ± 0.4	3.4 ± 0.3	0.5482
CVI	5.8 ± 3.3	5.4 ± 4.0	0.7081	6.5 ± 3.0	5.2 ± 3.5	0.1295	5.1 ± 2.6	5.3 ± 2.7	0.6920
